# A Triple-Mode Flexible E-Skin Sensor Interface for Multi-Purpose Wearable Applications

**DOI:** 10.3390/s18010078

**Published:** 2017-12-29

**Authors:** Sung-Woo Kim, Youngoh Lee, Jonghwa Park, Seungmok Kim, Heeyoung Chae, Hyunhyub Ko, Jae Joon Kim

**Affiliations:** 1 School of Electrical and Computer Engineering, Ulsan National Institute of Science and Technology, Ulsan 44919, Korea; karic525@unist.ac.kr (S.-W.K.); nailmong@unist.ac.kr (S.K.); laplume@unist.ac.kr (H.C.); 2 School of Energy and Chemical Engineering, Ulsan National Institute of Science and Technology, Ulsan 44919, Korea; duddh5438@unist.ac.kr (Y.L.); myidx1212@unist.ac.kr (J.P.)

**Keywords:** electronic skin, readout integrated circuit, sensor interface, wearable device, triple-mode, multi-purpose

## Abstract

This study presents a flexible wireless electronic skin (e-skin) sensor system that includes a multi-functional sensor device, a triple-mode reconfigurable readout integrated circuit (ROIC), and a mobile monitoring interface. The e-skin device’s multi-functionality is achieved by an interlocked micro-dome array structure that uses a polyvinylidene fluoride and reduced graphene oxide (PVDF/RGO) composite material that is inspired by the structure and functions of the human fingertip. For multi-functional implementation, the proposed triple-mode ROIC is reconfigured to support piezoelectric, piezoresistance, and pyroelectric interfaces through single-type e-skin sensor devices. A flexible system prototype was developed and experimentally verified to provide various wireless wearable sensing functions—including pulse wave, voice, chewing/swallowing, breathing, knee movements, and temperature—while their real-time sensed data are displayed on a smartphone.

## 1. Introduction

Wearable applications have evolved into various unobtrusive sensing device forms including wearable garment [1], watches [2], daily objects like automotive steering wheels [3]. Recently, skin-attachable device forms called electronic skins (e-skins) have been studied to improve body contact stability against various motion artifacts. Especially flexible e-skin materials provide valuable deformability and conformability on surfaces even with various topologies and geometries [4]. Many recent e-skin devices based on flexible films [5,6] or textiles [7] present ‘piezoresistive’ characteristics that their resistance varies with external static pressures. Another kind of e-skin devices which are mostly utilized for energy harvesting applications show a ‘piezoelectric’ characteristic the instant electrical charges occur in response to dynamic external pressures, which can be utilized to detect vocal-cord movement or heart rate [8]. Additionally, some e-skin devices are reported to have capability to detect temperature changes [9], and its wearing gives direct measurement of skin temperature. Another important trend of e-skin technologies is the multi-functional capability [10] that a single e-skin device can detect multiple sensing targets, which is valuable in artificial skin applications to mimic real human skins or tissues. 

Conventional e-skin studies have been focused on new device structures or material compositions, and their signal processing has been done by utilizing measurement equipment or simple readout interfaces like resistor dividers for resistance-to-voltage conversion. However, most e-skin devices operate in contact with the human body, so various noises might degrade or distort their detected signals [11]. Additionally, since they operate usually with batteries for power source, overall power consumption needs to be optimized by considering both their required performance level and implementation cost. Therefore, this work presents a triple-mode (priezoresistive, piezoelectric, pyroresistive) e-skin sensor interface including in-house multi-functional ferroelectric e-skin devices with fingertip skin-spired microstructure [12], where its readout integrated circuit (ROIC) is fabricated in a 0.18 μm CMOS process. The ROIC has a reconfigurable structure to provide three operation modes of P, Q, and T for the triple e-skin function. That is, the P mode gives the piezoresistive readout interface to cover various kinds of static pressures, the Q mode for the piezoelectric function detects instant device charges in response to external dynamic pressures, and the T mode detects temperature-induced resistance change in the pyroresistive operation. For sophisticated implementation of the ROIC, a linearization circuit for wide range operation of non-linear e-skin devices and a low-power oscillation-based detection circuit for pyroresistive devices with high resistance are proposed, and noise-enhancing circuits such as chopper stabilization [13] are included together. Furthermore, data converters including the analog-to-digital converter (ADC) are also integrated into the ROIC. For feasibility verification of the proposed triple-mode e-skin sensor interface, a flexible multi-functional e-skin module is fabricated and verified to provide six e-skin sensor operations on pulse wave, voice, swallowing, breathing, knee movements, and temperature while their real-time measured signals are wirelessly displayed on a smartphone.

The remainder of this paper is organized as follows: Section 2 presents the proposed multi-functional e-skin interface architectures. Section 3 contains detail designs of the reconfigurable ROIC. Section 4 shows experimental results. Finally, the conclusion is given in Section 5.

## 2. Triple-Mode E-Skin Integrated Electronic System

**Figure 1:**
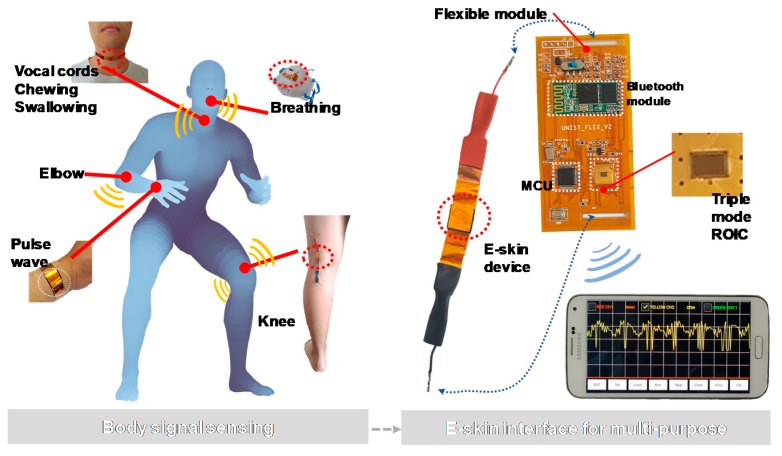
A triple-mode flexible e-skin sensor interface.

Figure 1 shows a triple-mode e-skin integrated electronic system whose triple-mode operations are implemented by utilizing polyvinylidene fluoride and reduced graphene oxide (PVDF/RGO) composite structures as e-skin sensing devices with three electrical properties. This reconfigurable system is applicable to mouth, chin, neck, elbow, wrist, knee, and other parts of human body, giving rise to fine tremors and motion. Signals detected from these body parts are electrically processed through single triple-mode reconfigurable ROIC, and then they can be wirelessly monitored in real time by including a Bluetooth module (HC-06), a microprocessor (MCU; ARM Coretex-M0), and a battery onto a flexible printed circuit board (PCB). The e-skin devices are characterized in three forms—priezoresistive, piezoelectric, and pyroresistive—according to external readout topologies, and each sensing property can be controlled by the amount of graphene in the composite process. This means that the e-skin device and the ROIC should be designed to be optimally correlated or reconfigurable for multiple desired e-skin sensing functions.

Therefore, the ROIC is proposed to have a reconfigurable structure for supporting three operational modes of the PVDF/RGO e-skin device. First, the ‘P’ mode provides a method to detect static pressures up to 20 kPa by utilizing the piezoresistive characteristic of the e-skin device for motion and pulse wave. Second, the ‘Q’ mode provides a method to process the piezoelectric characteristic and it mainly deals with dynamic pressure characteristics having frequency components. Third, the ‘T’ mode has high-resistance operating range and performs signal processing according to the pyroresistive input of resistance change caused by temperature change. The e-skin module is implemented to have a polyimide-based flexible PCB substrate and also designed to be easily worn on human body parts such as the neck, arms, and legs.

### 2.1. E-Skin Device Fabrication

**Figure 2:**
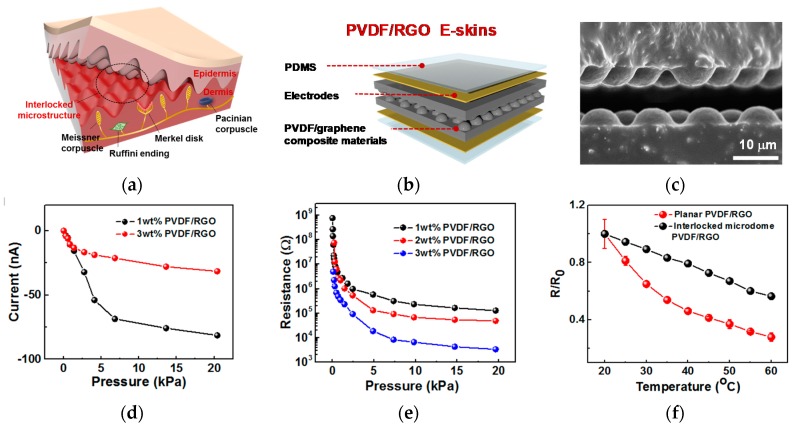
Design concept and electrical characteristic of PVDF/RGO e-skin device: (**a**) structure of fingertip skin; (**b**) bio-inspired e-skin device from fingertip skin; (**c**) micrograph of e-skin device; (**d**) piezoelectric characteristic; (**e**) piezoresistive characteristic; and (**f**) pyroresistive characteristic.

The PVDF/GO composite material is prepared by mixing PVDF and GO dispersed in DMF solvent at 70 °C for 2 h, followed by heat treatment in a vacuum oven. The PVDF/GO composite is made by removing residual DMF by heating to 70 °C in a vacuum oven. In addition, the PVDF/RGO composite material is subjected to heat treatment at 160 °C for a certain period of time in order to remove residual DMF in the composite material and to secure the conductivity of the composite material as shown in Figure 2b. In this process, the GO is reduced to RGO. In order to fabricate PVDF/RGO e-skins with a microdomain structure, a composite material solution is poured into a PDMS mold with a microdome surface. Heat treatment should be carried out in a vacuum oven. Two PVDF/RGO films fabricated through micromolding can be laminated on the surface of the microdome facing each other, and a copper or gold electrode can be deposited on the bottom and top layers to form an interlocking e-skin.

### 2.2. Electrical Characteristics of an E-Skin Device

An e-skin device consisting of a composite of PVDF/RGO was made by mimicking human epidermis. Figure 2a shows how human fingertip skin is an interlocked microstructure, in which the receptors that collect external pressure or temperature are gathered together. The e-skin device proposed in this paper also implements the functions of receptors to detect pressure and temperature of the skin in an interlocked structure. Figure 2b,c are magnified images of the e-skin device using PVDF/RGO and show the interlocked structure that is mimic structure. The electrical characteristics of e-skin devices made of PVDF/RGO composites have three characteristics such as receptor of fingertip skin: piezoelectric, piezoresistive, and pyroresistive. The piezoelectric property captures the characteristics of the conductivity of the material depending on the degree of mixing of RGO as shown in Figure 2d. Pyroresistive and piezoresistive properties reflect a change in resistance due to the skin simulating structure with an interlocked geometric structure depending on temperature and pressure. Figure 2e shows the curve of the resistance according to the associated pressure. Additionally, the linearity of the curve varies with the weight percent (wt %) of the RGO compound. Figure 2f shows a pyroresistive characteristic and resistance changes with a temperature change. Compared with conventional planar structures, the interlocked structure shows better linearity of temperature variation. In this way, the e-skin device has a variety of electrical properties to mimic fingertip skin, so optimized signal processing is required to detect each type of signal.

## 3. ROIC Implementation

### Triple-Mode Reconfigurable ROIC for an E-Skin Device

**Figure 3:**
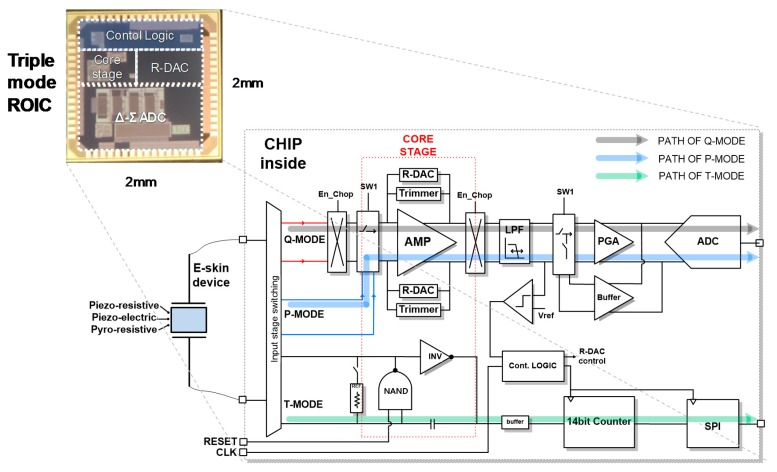
Block diagram of triple-mode reconfigurable readout integrated circuit (ROIC).

Unlike conventional readout circuits [14], the ROIC is designed to be reconfigurable so that it can support three characteristic mode of the PVDF/RGO e-skin device. Figure 3 shows the circuit diagram of a triple-mode reconfigurable ROIC, where triple modes of P, Q, and T are implemented to reuse common blocks, minimize the area, and minimize the power consumption. The P mode is implemented as a resistive sensor interface circuit using a differential amplifier and a resistive digital-to-analog converter (R-DAC). Differential amplifiers are used to linearize detection signals after comparing e-skin device with R-DAC. The Q mode acts as a differential charge amplifier which is similar to an integrator, reusing the P mode circuits. The T mode is a kind of an oscillator-based resistance to digital converter to support high-resistance region over tens of mega-ohms. Digital outputs in these triple modes are transferred to the MCU through the serial peripheral interface (SPI), and then wirelessly delivered to the smartphone through the Bluetooth.

**Figure 4:**
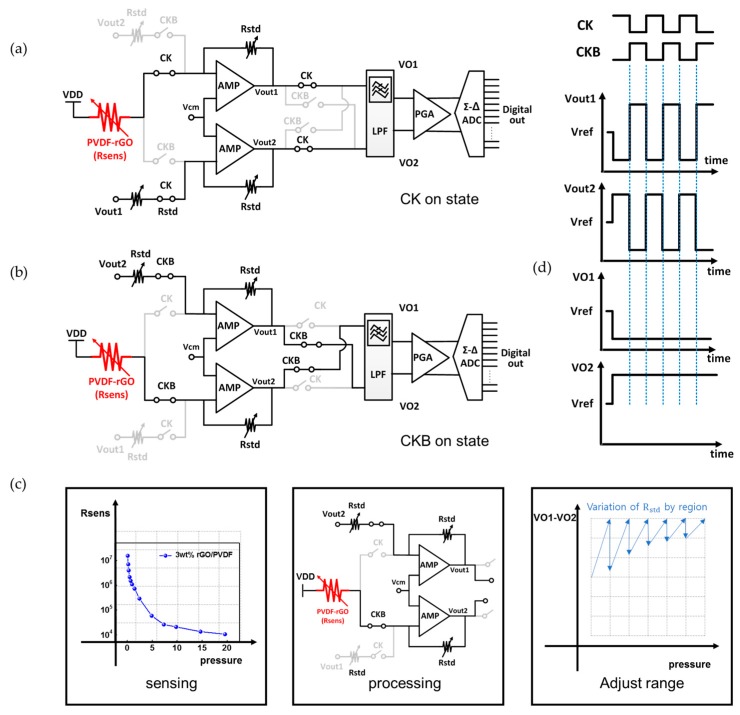
Detailed circuit operation of P mode for static pressures: (**a**) active path when CK is on state; (**b**) active path when CKB on state; (**c**) linearization process; and (**d**) transient node voltages.

Figure 4 shows detailed circuit operation of the P mode which senses the static pressure. The P mode has a reconfigured signal-processing structure that uses a differential amplifier and a programmable gain amplifier (PGA). In particular, the chopper stabilization is applied around the first differential amplifier to suppress low frequency noises, including flicker noises, while performing single-ended to differential signal conversion. Depending on the chopper clock, the input path to the e-skin device changes alternately. As shown in Figure 4a,b, the chopper stabilization with CK and CLKB connects the e-skin device to V_out1_ or V_out2_ via R_std_. The R_std_ is the resistance value of the R-DAC which is adjusted by the comparator and the control logic so that the P mode can detect wide range of resistances. The nodal analysis of Figure 4a around the differential amplifier can be written as

(1) $$\frac{\mathrm{VDD}-V_{\mathrm{cm}}}{R_{\mathrm{sens}}}=\frac{V_{\mathrm{cm}}-V_{\mathrm{out}1}}{R_{\mathrm{std}}}$$

(2) $$\frac{V_{\mathrm{out}1}-V_{\mathrm{cm}}}{R_{\mathrm{std}}}=\frac{V_{\mathrm{cm}}-V_{\mathrm{out}2}}{R_{\mathrm{std}}}$$

From Equations (1) and (2), the differential amplifier outputs can be derived by

(3) $$V_{\mathrm{out}1}=V_{\mathrm{cm}}-\frac{R_{\mathrm{std}}}{R_{\mathrm{sens}}}\left(\mathrm{VDD}-V_{\mathrm{cm}}\right)$$

(4) $$V_{\mathrm{out}2}=V_{\mathrm{cm}}+\frac{R_{\mathrm{std}}}{R_{\mathrm{sens}}}\left(\mathrm{VDD}-V_{\mathrm{cm}}\right)$$

When CKB is on state as in Figure 4b, the same equation is derived like Equations (3) and (4). Since the e-skin device resistance is inversely proportional to its pressure input, the differential output of Equations (3) and (4) can have a linear relationship with the pressure. For wide coverage of R_sens_, R_std_ is tuned depending on the region not to saturate the amplification, and the differential output would have linear relationship with the pressure input as shown in Figure 4c. Transient waveforms of the differential outputs are also shown in Figure 4d. When an e-skin device is worn on human body, in this linear relationship process based on the resistance ratio, the resistance variation of the e-skin device from bending characteristic is minimized by adjusting range in R-DAC. In this way, the P mode converts single-ended signals of the e-skin device into differential signals so that the pseudo-differential structure and the chopper stabilization can increase the noise immunity considerably. 

**Figure 5:**
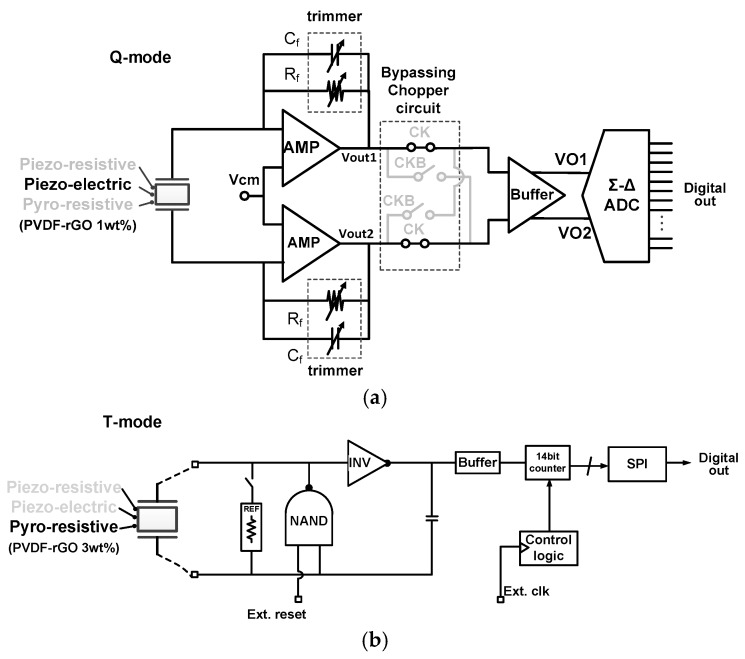
Circuit-level implementations for (**a**) Q mode and (**b**) T mode.

In the Q mode, the e-skin device is connected to two input nodes in parallel as shown in Figure 5a, and the differential–amplifier pair is reconfigured to act as an integrator by changing its internal paths. The e-skin device generates charges from dynamic pressures, and the integrator operation convert the input charge to the differential output voltage. The detected signal passing through the buffer is inserted to the ADC, and then converted to the digital output.

**Figure 6:**
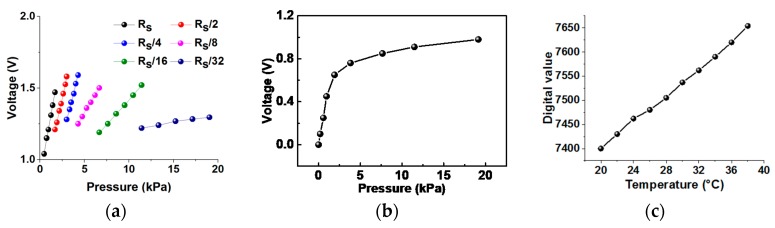
Sensor readout characteristics after triple-mode signal process: (**a**) P mode; (**b**) Q mode; (**c**) T mode.

In the T mode for temperature sensing, the e-skin device characteristic is located in high-resistance range over tens of mega-ohms when pressure is not applied as shown in Figure 2e. Thus, instead of using conventional voltage-converting methods, an oscillator-based readout is adopted to generate frequency displacements according to resistance changes, where the frequency displacement is converted into a digital value through the counting logic using an external clock. The oscillator consists of a NAND gate, an inverter, and a RC network. When the reset signal is ‘H’, the oscillator repeats charging and discharging whose period is decided by time constant of RC. The following counting operation converts the period or the frequency to a digital output which explains temperature sensing of the e-skin device. Figure 6 presents measured characteristic curves of these three operation modes.

Since two interfaces of the P and T modes are commonly for resistive sensors, they might be implemented into single readout circuit. However, the proposed reconfigurable interface tried to provide separate readout circuits for two resistive detections of static pressure and temperature. In cases of temperature without external pressures, the resistance variation range is normally limited to a high-resistance region above mega-ohms so that the low-power oscillator-based readout method (T mode) is adopted. In cases of static pressure, we adopted the voltage-based readout method (P mode) to support wide range of resistance range from kilo-ohms to mega-ohms, also providing good noise immunity against various environmental noises, including motion artifacts. Whereas, if the P mode is used for the temperature with high characteristic resistance, its readout signal becomes noisy because its operating current for the voltage conversion drops below micro-Amperes.

## 4. Experimental Results

### 4.1. Flexible E-Skin Sensor Module

A flexible e-skin sensor module was implemented to provide real-time detections of human body signals by wearing the e-skin device as shown in Figure 1. The triple-mode reconfigurable ROIC is directly mounted on the flexible sensor module in the form of a chip on board (COB). The module size is 3.9 × 8.1 cm and its weight is 10.9 g, including both the e-skin device and battery. Two PVDF/RGO composites of 1 wt % and 3 wt % were applied for the e-skin sensor. The 1 wt % PVDF/RGO sample gave better piezoelectric responses with more charge generation and the 3 wt % sample provided better piezoresistive responses with lower characteristic resistance, whose sizes are 2.5 × 2.5 cm and 1.2 × 1.2 cm respectively. They attached two copper electrodes on both sides using silver paste and annealed them at 90 °C for 1 h. The flexible PCB that is made of polyimide-based flexible substrate improved flexibility by placing margins in the conductive layer to be worn on the human body. This blank area increases the module robustness from the bending. The input and output terminals were made by constructing holders such that it can connect with the e-skin device stably. Silicon molding was applied to sensitive input terminals and surfaces to prevent disconnection even if the PCB substrate is bent. Output signals are transmitted through the Bluetooth to an Android-based mobile platform where wireless real-time monitoring is provided by connecting to analytical applications.

### 4.2. Measurement Results of E-Skin Sensor Interfaces

**Figure 7:**
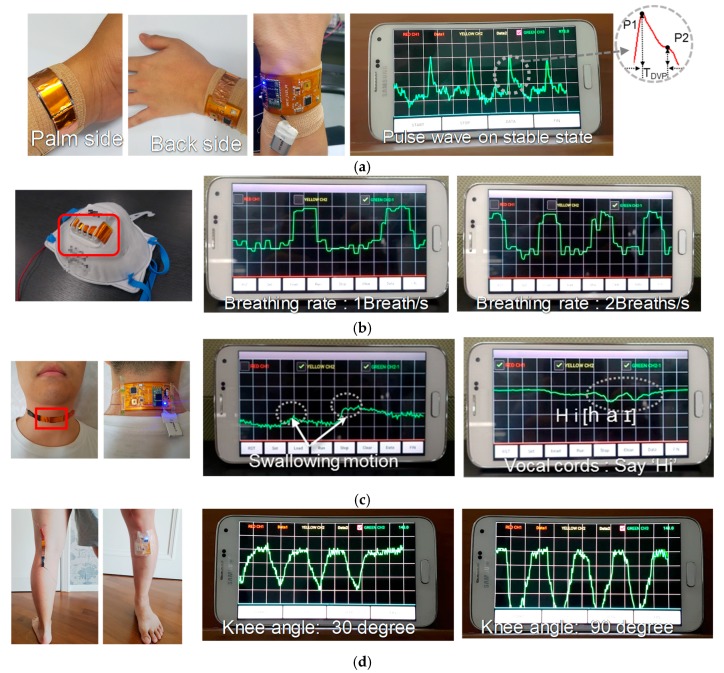
Experimental results of P-mode e-skin sensor interface: (**a**) pulse wave; (**b**) breathing; (**c**) swallowing and movement of vocal cords; (**d**) knee angle.

The P mode can detect static pressures such as motion of the human body and pulse waves of the radial artery. In the case of the pulse wave, Figure 7a shows its wearing method and monitored results on the android-based smartphone while the flexible integrated module was worn around the wrist and the e-skin device was located and bent inward to contact the radial artery. The measured pulse-wave waveform on the smartphone was obtained from a 30-year-old male; P1, P2, and ΔT_DVP_ are prominent parts of the radial pulse wave. The ratio of the peak pressure to the inflation pressure and the time difference which is the radial artery augmentation index (AI_r_), give valuable information on cardiovascular status. The indices of the enlarged pulse wave in Figure 7a can be calculated as AI_r_ = P2/P1 = 0.45 and ΔT_DVP_ = 0.15 s, which is a measure close to the radial augmentation indices of 20–30 years old [15]. Figure 7b shows monitoring results of breathing intensity and respiration rate for exhalation. The e-skin device is installed inside a mask, and measured waveforms show intensity and rate of exhalation when the e-skin mask is worn. The peak pressure corresponds to the inspiration of a person at about 1 to 3 kPa and falls within the detection range of the e-skin device and the triple-mode system [16]. Figure 7c shows experimental results on vocal cords and uvula. The e-skin module was worn around the neck, and the e-skin device was located on the vocal cords or uvula. The e-skin device senses movement or vibration of the vocal cords during speaking. Measure waveforms show vibration of the vocal cords when someone says “Hi” and it shows the swallowing motion of the uvula when the device is located at 1 cm above the vocal cords [17,18,19]. The e-skin sensor signal changes according to vocalization and accent of the vowel. Figure 7d shows experimental results on folding motion of the knee. The e-skin device was attached to the back of the knee and the sensor module in patch form was attached to lower part of the knee to prevent any hindering from leg motions. Measured waveforms on the smartphone are shown to vary with bending angle of the knee [20,21]. As the bending angle increases, the slope of the curve becomes steeper and deeper. It can be attached to other parts of the body like the elbow, where similar bending movements occur.

**Figure 8:**
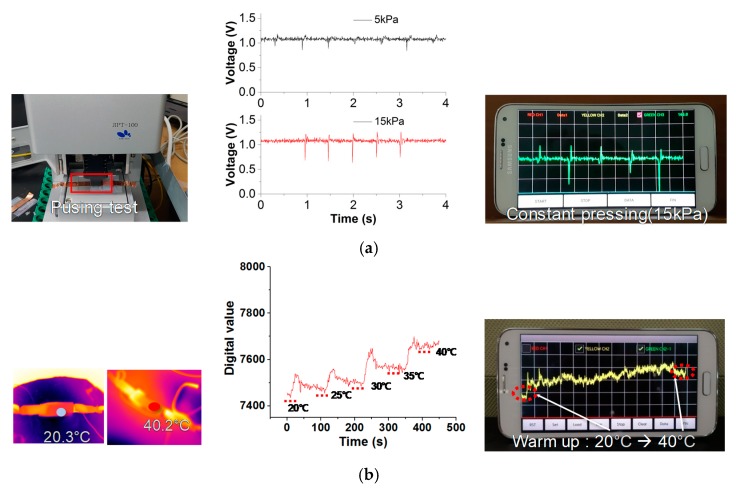
Experimental results of multi-mode e-skin sensor module: (**a**) Q mode; (**b**) T mode.

The Q mode was demonstrated by utilizing the pushing tester (JIPT 110 of JUNIL TECH) to evaluate detection capability on constant pressing as in Figure 8a. When periodic constant pressure was applied, the output signal was measured at the ROIC and also monitored on the smartphone. The T mode provides temperature measurement as shown in Figure 8b. The e-skin device was heated and measured while the temperature sweeps 20 °C to 40 °C with intervals of 5 °C. The e-skin device has pyroresistive characteristics with a negative temperature coefficient. 

E-skin devices are based on the composite material which has conductivity, flexibility, and robustness, but they have been mostly developed without sufficient considerations on their readout interface and signal processing. Therefore, this study intensified their readout interfaces with wireless monitoring platform and flexible e-skin sensor module implementation for comfortable patch-type sensing applications. We implemented the triple-mode reconfigurable interface to detect body motions, pulse wave, dynamic pressure, and temperature variation. In particular, the detection and analysis of the radial pulse waves suggested the possibility of managing and monitoring symptoms of hypertension. Through the detection of vocal-cords movement and food intake, we were able to monitor the pharyngeal muscle and to perform mobility tests with breathing and knee-folding demonstrations [22]. Current multi-functionality of e-skin sensor interfaces is limited to separate applications of each mode, where simultaneous detection responses of piezoresistive and pyroresistive modes are not distinguishable. A further step would be to achieve the simultaneous multi-functionality of the e-skin sensor interface and also to analyze the motion of the body in more detail by developing frequency-domain analyses and pattern-recognition algorithms. Additionally, the P mode for the static pressure might be affected by the temperature, and another further study would try to compensate it by making additional algorithms, considering that static-pressure response time is much faster than the temperature response time.

## 5. Conclusions

This work presented a triple-mode flexible e-skin sensor system which includes a flexible PVDF/RGO sensor device, a reconfigurable ROIC, and a mobile interface. Based on measured multi-functional characteristics of an in-house e-skin device, the ROIC was also optimally designed to support triple-mode interfaces for piezoelectric, piezoresistive, and pyroresistive sensing targets. Seven wearable application experiments showed feasibility of the proposed e-skin sensor interface for multiple sensor applications, where flexible sensor modules were properly adapted to provide comfortable skin-attached measurements depending on their target locations.

## References

[1] Zheng Y.-L., Ding X.-R., Poon C.C.P., Lo B.P.L., Zhang H., Zhou X.-L., Yang G.-Z., Zhao N., Zhang Y.-T. (2014). Unobtrusive Sensing and Wearable Devices for Health Informatics. IEEE Trans. Biomed. Eng..

[2] Woo S.H., Choi Y.Y., Kim D.J., Bien F., Kim J.J. (2014). Tissue-Informative Mechanism for Wearable Non-invasive Continuous Blood Pressure Monitoring. Sci. Rep..

[3] Choi S., Kim D.J., Choi Y.Y., Park K., Kim S.-W., Woo S.H., Kim J.J. (2017). A Multi-Sensor Mobile Interface for Industrial Environment and Healthcare Monitoring. IEEE Trans. Ind. Electron..

[4] Wang H., Dong L., Zhang H., Yu R., Pan C., Wang Z.L. (2015). Recent progress in electronic skin. Adv. Sci..

[5] Lipomi D.J., Vosgueritchian M., Tee B.C.-K., Hellstrom S.L., Lee J.A., Fox C.H., Bao Z. (2011). Skin-like pressure and strain sensors based on transparent elastic films of carbon nanotubes. Nat. Nanotech..

[6] Park J., Lee Y., Hong J., Lee Y., Ha M., Jung Y., Lim H., Kim S.Y., Ko H. (2014). Tactile-direction-sensitive and stretchable electronic skins based on human-skin-inspired interlocked microstructures. ACS. Nano.

[7] Lee T., Lee W., Kim S.-W., Kim J.J., Kim B.-S. (2016). Flexible Textile Strain Wireless Sensor Functionalized with Hybrid Carbon Nanomaterials Supported ZnO Nanowires with Controlled Aspect Ratio. Adv. Funct. Mater..

[8] Kang D., Pikhitsa P.V., Choi Y.W., Lee C., Shin S.S., Piao L., Park B., Suh K.-Y., Kim T.-I., Choi M. (2014). Ultrasensitive mechanical crack-based sensor inspired by the spider sensory system. Nat. Lett..

[9] Lee J.S., Shin K.-Y., Cheong O.J., Kim J.H., Jang J. (2015). Highly sensitive and multifunctional tactile sensor using free-standing ZnO/PVDF thin film with graphene electrodes for pressure and temperature monitoring. Sci. Rep..

[10] Hammock M.L., Chortos A., Tee B.C.-K., Tok J.B.-H., Bao Z. (2013). 25th Anniversary article: The evolution of electronic skin (E-skin): A brief history, design considerations, and recent progress. Adv. Mater..

[11] Lee K., Choi Y.Y., Kim D.J., Chae H.Y., Park K., Oh Y.M., Woo S.H., Kim J.J. (2017). A wireless ExG interface for patch-type ECG holter and EMG-controlled robot hand. Sensors.

[12] Park J., Kim M., Lee Y., Lee H.S., Ko H. (2015). Fingertip skin-inspired microstructured ferroelectric skins discriminate static/dynamic pressure and temperature stimuli. Sci. Adv..

[13] Enz C.-C., Temes G.-C. (1996). Circuit Techniques for Reducing the Effects of Op-Amp Imperfections: Autozeroing, Correlated Double Sampling, and Chopper Stabilization. Proc. IEEE.

[14] Massarotto M., Carlosena A., Lopez-Martin A.J. (2008). Two-stage differential charge and transresistance amplifiers. IEEE Trans. Instrum. Meas..

[15] Nichols W. (2005). Clinical measurement of arterial stiffness obtained from noninvasive pressure waveforms. Am. J. Hypertens..

[16] Cournand A., Motley H., Werko L., Richards J.R.D. (1947). Physiological studies of the effects of intermittent positive pressure breathing on cardiac output in man. Am. J. Physiol..

[17] Liu Q., Chen J., Li Y., Shi G. (2016). High-performance strain sensors with fishscale-like graphene-sensing layers for fullrange detection of human motions. ACS Nano.

[18] Wang X., Gu Y., Xiong Z., Cui Z., Zhang T. (2014). Silk-molded flexible, ultrasensitive, and highly stable electronic skin for monitoring human physiological signals. Adv. Mater..

[19] Fontana J., Sazonov E., Sazonov E., Neuman M. (2014). Wearable Sensors.

[20] Yao S., Zhu Y. (2014). Wearable multifunctional sensors using printed stretchable conductors made of silver nanowires. Nanoscale.

[21] Yamada T., Hayamizu Y., Yamamoto Y., Yomogida Y., Najafabadi A., Futaba D., Hata K. (2011). A stretchable carbon nanotube strain sensor for human-motion detection. Nat. Nanotechnol..

[22] Mishra S., Norton J., Lee Y., Lee D.S., Agee N., Chen Y., Chun Y., Yeo W.-H. (2017). Soft, conformal bioelectronics for a wireless human-wheelchair interface. Biosens. Bioelectron..

